# A Belief Two-Level Weighted Clustering Method for Incomplete Pattern Based on Multiview Fusion

**DOI:** 10.1155/2022/2895338

**Published:** 2022-11-30

**Authors:** Zong-fang Ma, Hui-xuan Zhao, Lei-hua Li, Lin Song

**Affiliations:** ^1^College of Information and Control Engineering, Xi'an University of Architecture and Technology, Xi'an 710311, China; ^2^Unmanned System Research Institute, Northwestern Polytechnical University, Xi'an 710129, China

## Abstract

Incomplete pattern clustering is a challenging task because the unknown attributes of the missing data introduce uncertain information that affects the accuracy of the results. In addition, the clustering method based on the single view ignores the complementary information from multiple views. Therefore, a new belief two-level weighted clustering method based on multiview fusion (BTC-MV) is proposed to deal with incomplete patterns. Initially, the BTC-MV method estimates the missing data by an attribute-level weighted imputation method with k-nearest neighbor (KNN) strategy based on multiple views. The unknown attributes are replaced by the average of the KNN. Then, the clustering method based on multiple views is proposed for a complete data set with estimations; the view weights represent the reliability of the evidence from different source spaces. The membership values from multiple views, which indicate the probability of the pattern belonging to different categories, reduce the risk of misclustering. Finally, a view-level weighted fusion strategy based on the belief function theory is proposed to integrate the membership values from different source spaces, which improves the accuracy of the clustering task. To validate the performance of the BTC-MV method, extensive experiments are conducted to compare with classical methods, such as MI-KM, MI-KMVC, KNNI-FCM, and KNNI-MFCM. Results on six UCI data sets show that the error rate of the BTC-MV method is lower than that of the other methods. Therefore, it can be concluded that the BTC-MV method has superior performance in dealing with incomplete patterns.

## 1. Introduction

In the information era, data have abundant research value, but collecting complete data is significantly difficult. In the collection process, the reasons for missing data are varied, including subjective and objective factors, such as equipment malfunction, personnel operation error, false memory, and partial rejection by the respondents [1]. Missing data, also called an incomplete pattern, is a common phenomenon in practical applications. A survey shows that 45% of data sets in the UCI machine learning repository, which covers many fields, are incomplete [2]. Deletion and imputation methods are commonly used to deal with missing data. Deleting incomplete patterns is an easy method, which is acceptable when the incomplete pattern accounts for less than 5% of the whole data set [3]. The imputation method, which replaces missing values with estimations, is a popular method for dealing with incomplete patterns [4]. For instance, the KNN technology and its derivatives have been used in many application fields because of their strong operability [5–7].

A number of imputation methods based on the KNN technology have been proposed [8–10]. In the early method, the average value of the k-nearest neighbor about the incomplete pattern was used to express the missing value [9]. In addition, the imputation methods of integrating KNN and other technologies were proposed by some researchers [11–13]. For example, an adaptive imputation method for missing values, which uses KNN and self-organizing map (SOM) based on belief function theory, is proposed in [11]. In this method, the uncertainty caused by the missing data is represented. The linear local approximation method is presented, which uses the KNN with optimal weights obtained by local linear reconstruction technology to estimate the missing values [13]. The estimated values obtained by the traditional KNN based on a single view are globally optimum but may not be locally optimum. Therefore, the imputation method based on a single view may decrease the accuracy of clustering methods.

Clustering is an important task of pattern recognition and machine learning, which divides objects into different clusters based on the similarity between patterns [14]. Hard clustering and fuzzy clustering methods have been used in many fields by their universality [15–17], but the clustering methods based on a single view ignore the information from multiple views [18]. Compared with the single-view clustering method, the clustering method based on multiple views explores the complementary information of each view, which can improve the accuracy of the clustering result [19, 20]. Recently, multiview clustering has become a popular research topic [21, 22]. A collaborative multiview clustering method is proposed in [23] to overcome disagreement between the views, the different properties, and scales of views. The weights that represent the importance of views and features are proposed in [24]; an objective function is designed to express the heterogeneity of different views and the consistency across views during iterations. Jiang et al. [25] proposed the multiview FCM clustering algorithm with views and feature weights based on collaborative learning; this method can exclude irrelevant components in the clustering procedure, which increases the precision of the clustering results. In addition, multiview spectral clustering methods have been studied recently. The spectral clustering algorithms consist of two steps as follows: learning the similarity graphs from instances and obtaining the clustering result based on spectral clustering. Tang et al. [26] proposed a unified one-step multiview spectral clustering method (UOMvSC). In order to obtain the clustering results, the UOMvSC method combined the multiview embedding matrices and graphs into a unified graph. A joint affinity graph for multiview clustering is proposed in [27]; the diversity regularization term is designed to learn the different weights of diverse views. Zheng et al. [28] proposed a novel multiview clustering method that integrates within-view partial graph learning, cross-view partial graph fusion, and cluster structure recovery. However, most of the clustering methods for incomplete patterns are based on single-view, and the clustering results are not accurate enough. In addition, to our knowledge, there is little research on the multiview imputation method, although researchers have proposed numerous methods to improve the accuracy of the estimation.

In this paper, we develop a belief two-level weighted clustering method for incomplete patterns based on multiview fusion (BTC-MV). The main contributions of this work are summarized as follows:

1. Attribute-level weighted imputation strategy for incomplete patterns: In this strategy, the variance of each attribute, which is called attribute weight, is used to reflect the importance. The weighted attribute is used in searching for KNN of the incomplete pattern based on multiple views.
2. View-level weighted fusion strategy based on belief function theory: The view-level weights are obtained by optimizing the new objective function of the clustering method based on multiple views. They are regarded as the discounted factors in the belief fusion, which represent the importance of the evidence from different view spaces.
3. To the best of our knowledge, the belief two-level weighted clustering method for incomplete patterns based on multiview fusion is proposed for the first time. Compared with other state-of-the-art methods, the BTC-MV method performs better in multiview clustering for incomplete patterns.

The rest of this paper is organized as follows: In Section 2, we introduce related work on missing data classification methods and the basics of belief function theory. The details of the belief clustering method for incomplete patterns based on multiview fusion are shown in Section 3. In Section 4, we compare the BTC-MV method with other state-of-the-art methods on six UCI data sets. Finally, the conclusion is drawn in Section 5.

## 2. Related Work

### 2.1. Classification of the Missing Data

According to the missing mechanism, the incomplete pattern can be divided into missing completely at random (MCAR), missing at random (MAR), and missing not at random (MNAR) [29]. Wang et al. [30] proposed a query algorithm based on the Spark framework to handle query problems with incomplete data sets. The clustering method for incomplete patterns includes the imputation of the missing data and the clustering for data sets.

In addition to the abovementioned imputation method based on the KNN technology, the mean imputation (MI) and fuzzy c-means imputation (FCMI) methods have obtained significant research progress [31–33]. In MI [34], the missing data are estimated by the mean value or mode of the corresponding attribute, and it is used for the data sets with a similar attribute distribution in each category. However, the estimations of the same attribute in different incomplete patterns are equal. In FCMI [33], the estimations are calculated by the clustering centers and the distance between the centers and the patterns. However, the performance of this imputation strategy depends on initial conditions.

The clustering algorithm is applied to partition the data set into several clusters, and it has been widely used in various fields. A cluster-based information retrieval approach was proposed in [35]. The k-means clustering method and frequent closed item set mining were combined to extract clusters of documents and find frequent terms. The clustering method and pattern mining algorithm were integrated to search for the most relevant object from a clustered set of objects [36]. The space-time series clustering methods, such as hierarchical, partitioning-based, and overlapping clustering methods were used in big urban traffic data sets [37]. In addition to the single-view clustering methods described above, many researchers have extended the single-view clustering methods to multiview clustering methods. A multiview FCM clustering method based on the collaborative learning was proposed in [38]; it included a single-view partition process and a collaborative step to share information between different views. Wang and Chen [39] proposed a multiview fuzzy clustering method with minmax optimization. The multiview clustering method can integrate the information from different views.

In recent years, with the development of the neural networks, many models based on deep learning have been built to classify incomplete data sets [40–42]. In [41], a multivariate time series generative adversarial network is proposed for multivariate time series imputation, which improves the imputation performance. However, the performance of the deep learning classification models depends on large data sets. When the data set is small, the model cannot be stable.

### 2.2. Basics of the Belief Function Theory

The belief function theory is called evidence theory or Dempster–Shafer theory (DST), which is a classic theoretical framework used in probabilistic reasoning [43, 44]. The belief function theory can generate a belief mass by fusing the useful evidence from independent sources, which is used in many fields [45, 46]. In this theory, the discernment framework consists of finite, mutually exclusive, and complete elements of the problem under study, and it is represented by Ω={*ω*_1_, *ω*_2_,…, *ω*_*c*_}. The power-set of the discernment framework Ω expresses the uncertainty, which is denoted as 2^Ω^. The basic belief assignment (BBA) is a function *m*(∙) from 2^Ω^ to [0, 1], which satisfies the following conditions:

(1) $$\begin{array}{c} \left\{\begin{array}{c} \underset{A\in 2^{\Omega }}{\sum }m\left(A\right)\\ m\left(\emptyset \right)=0 \end{array}\right.\text{,} \end{array}$$

where *m*(*A*) expresses the probability of the evidence supporting proposition *A* but does not support the occurrence of any true subset of *A*. All elements that satisfy *A* ∈ 2^Ω^ and *m*(*A*) > 0 are called focal elements of *m*(∙).

The outputs of classifiers or fuzzy clustering methods indicate the extent of the corresponding evidence that supports different classes. The DS fusion theory [47, 48] is used in many fields because it can integrate the evidence from many independent sources by its commutative and associative properties. The fusion strategy of the evidence from different independent sources *m*_1_(∙) and *m*_2_(∙) at the discernment framework 2^Ω^ is shown as follows:

(2) $$\begin{array}{c} m\left(A\right)=\left\{\begin{array}{cc} \frac{\underset{B\cap C=A}{\sum }m_{1}\left(B\right)\;∙\;m_{2}\left(C\right)}{1-K} & \;A\neq \emptyset ,\forall A\in 2^{\Omega }\\ 0 & \;A=\emptyset \end{array}\right.\text{,} \end{array}$$

where$K=\underset{B\cap C=\emptyset }{\sum }m_{1}\left(C\right)∙m_{2}\left(B\right)$ indicates the conflict belief mass between evidence from different sources. However, the result of the DS fusion theory is unreasonable when the conflict between evidence from different sources is significantly high. Therefore, a series of methods are proposed to solve the abovementioned problems, such as a fusion strategy proposed by Dubois and Prade in [49] and PCR6 rules based on the proportional conflict redistribution [50].

## 3. Clustering Method for Incomplete Pattern

**Figure 1:**
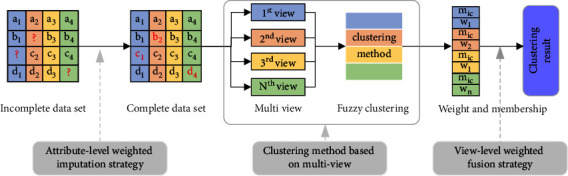
The flowchart of the BTC-MV method.

We propose the BTC-MV method to decrease the error rate of the clustering method in incomplete patterns, where data are randomly missing or unobserved. The flowchart of the BTC-MV method is presented in Figure 1. First, an attribute-level weighted imputation strategy is proposed to estimate the missing or unobserved value in the data set *X*. In this step, the variance of each attribute in the data set *X* is calculated and regarded as the weight of the KNN and the missing values are estimated by the KNN. Second, a fuzzy C-means clustering method based on multiple views is proposed to cluster the complete data set with estimated values, and the membership values and the weight of multiple views are submitted to a view-level weighted fusion strategy to get precise results. Third, the BTC-MV method uses the weight of multiple views as the discounted factors, and a belief fusion strategy is proposed to fuse the membership values of the pattern in different views. Finally, the clustering results are obtained. The details of the BTC-MV method are shown as follows.

### 3.1. Attribute-Level Weighted Imputation Strategy

Here, all attributes in data set *X* are divided into *N* views. *X*_*i*_^*μ*^ expresses the feature matrix of the pattern *x*_*i*_ under the view space *μ*. We assume that some attributes of pattern *x*_*i*_ are unobserved, because the clustering method for incomplete patterns is our research topic.

In the BTC-MV method, the attribute-level weighted imputation strategy based on the KNN is proposed to estimate the missing value. First, we calculate the variance of each attribute in the data set *X*, as shown in equation (3), which expresses the importance of different attributes in *X*. A bigger variance indicates a larger difference between all instances in the attribute space, so the estimation calculated by k-nearest instances is more accurate. Then, the weighted KNN method is proposed to search for the top-k-nearest neighbors of *y*_*i*_^*μ*^ in the view space *μ*; the distance between the complete pattern *𝓍*_*i*_^*μ*^ and the incomplete pattern *y*_*i*_^*μ*^ is shown in equation (4). The variance of the attribute is regarded as the weight of distance between complete patterns and incomplete patterns. According to the weighted distance, we obtain K neighbors closest to the incomplete pattern and estimate the missing value. Finally, the estimated value of the missing data is calculated by equation (5). The imputation strategy is shown in Algorithm 1, which introduces the variance of different attributes in *X* to estimate the missing data and improve the precision of the estimation.

(3) $$\begin{array}{c} \begin{array}{c} S_{p}^{\mu }=\frac{\sum {\left(x_{p}^{\mu }-{\bar{x}}_{p}^{\mu }\right)}^{2}}{p-1} \end{array}\text{,} \end{array}$$

(4) $$\begin{array}{c} d_{ip}=\sqrt{\overset{n_{\mu }}{\underset{p=1}{\sum }}{S_{p}^{\mu }\left(y_{ip}^{\mu }-x_{ip}^{\mu }\right)}^{2}}\text{,} \end{array}$$

(5) $$\begin{array}{c} {\hat{y}}_{ip}^{\mu }=\frac{\sum_{i=1}^{K}x_{ip}^{\mu }}{K}\text{,} \end{array}$$

where *𝓍*_*𝓅*_^*μ*^ is the *𝓅*th attributes of the data set *X* in the view space *μ*, *p* is the number of the patterns in the view space *μ*, *𝓃*_*μ*_ is the number of the attributes in view space *μ*, *S*_*𝓅*_^*μ*^ is the variance of attribute *𝓅*, and it is normalized as the weight of attribute *𝓅* under the view space *μ*. *𝓎*_*i*_ is an incomplete pattern with *t* unobservable attributes. *𝓍*_*i𝓅*_^*μ*^ is the complete sample belonging to class *ω*_*c*_ under the view space *μ*. *d*_*i𝓅*_ denotes the weighted distance between *𝓎*_*i𝓅*_^*μ*^ and *𝓍*_*i𝓅*_^*μ*^. ${\hat{𝓎}}_{i𝓅}^{\mu }$ is the estimated value of the incomplete pattern *𝓎*_*i*_^*μ*^ in the attribute space *𝓅*. ∑_*i*=1_^*K*^*𝓍*_*i𝓅*_^*μ*^ is the sum of the top-K nearest neighbors for *𝓎*_*i*_^*μ*^.

### 3.2. Clustering Method Based on Multiple Views

After the attribute-level weighted imputation method based on multiple views, the data set *X* is regarded as a complete data set with estimations. The fuzzy C-means clustering method based on multiple views is conducted on the complete data set *X*, which is shown in Algorithm 2. In each view, we calculate the clustering centers, the membership values, and the view weights. The objective function of the clustering method based on multiple views is shown in the following equation:

(6) $$\begin{array}{c} J\left(W,M,V\right)=\overset{N}{\underset{\mu =1}{\sum }}w_{\mu }\overset{p}{\underset{i=1}{\sum }}\overset{C}{\underset{c=1}{\sum }}\overset{n_{\mu }}{\underset{p=1}{\sum }}m_{ic}^{\beta }d^{2}\left(x_{ip}^{\mu },v_{cp}^{\mu }\right)+\gamma \overset{N}{\underset{\mu =1}{\sum }}w_{\mu }\mathrm{lg}\left(w_{\mu }\right)\text{,} \end{array}$$

s.t.

(7) $$\begin{array}{c} \left\{\begin{array}{c} \overset{C}{\underset{c=1}{\sum }}m_{ic}=1,i=1,\ldots ,p,\\ \overset{N}{\underset{\mu =1}{\sum }}w_{\mu }=1, \end{array}\right. \end{array}$$

where *β* is the weight exponent that determines the fuzziness of the clustering result, *m*_*ic*_ is the membership value of the *i*th pattern *x*_*i𝓅*_ to the *c*th cluster center *v*_*c𝓅*_, and *w*_*μ*_ is the weight of the *μ* th view. *d*^2^(*𝓍*_*i𝓅*_^*μ*^, *v*_*c𝓅*_^*μ*^) expresses the Euclidean distance between *x*_*i𝓅*_ and *v*_*c𝓅*_ in the view space *μ*.

The optimal values of the multiview clustering method are obtained by minimizing the objective function by iterative optimization. In general, the optimal values are derived by setting the partial derivatives of the objective function to zero. According to the Lagrangian multiplier method, the Lagrangian function of the objective function *J* under the constraints of equation (7) is shown in the following equation:

(8) $$\begin{array}{c} L\left(W,M,V\right)=\overset{N}{\underset{\mu =1}{\sum }}w_{\mu }\overset{p}{\underset{i=1}{\sum }}\overset{C}{\underset{c=1}{\sum }}\overset{n_{\mu }}{\underset{p=1}{\sum }}m_{ic}^{\beta }d^{2}\left(x_{ip}^{\mu },v_{cp}^{\mu }\right)+\gamma \overset{N}{\underset{\mu =1}{\sum }}w_{\mu }\mathrm{lg}\left(w_{\mu }\right)\\ +\overset{p}{\underset{i=1}{\sum }}\lambda_{i}\left(\overset{C}{\underset{c=1}{\sum }}m_{ic}-1\right)+\phi \left(\overset{N}{\underset{\mu =1}{\sum }}w_{\mu }-1\right)\text{,} \end{array}$$

where *λ*_*i*_ and *ϕ* are the Lagrangian multipliers.

The optimal values of the objective function *J*, such as the cluster center *v*_*c𝓅*_^*μ*^, the weight of the view *w*_*μ*_, and the membership value *m*_*ic*_, are obtained by calculating partial derivatives of the function *L*, which are shown in the following equations:

(9) $$\begin{array}{c} v_{cp}^{\mu }=\frac{\sum_{i=1}^{p}m_{ic}^{\beta }x_{ip}^{\mu }}{\overset{p}{\underset{i=1}{\sum }}m_{ic}^{\beta }}\text{,} \end{array}$$

(10) $$\begin{array}{c} w_{\mu }=\frac{\exp\;\left(-F^{\mu }/\gamma \right)}{\overset{N}{\underset{\mu =1}{\sum }}\exp\;\left(-F^{\mu }/\gamma \right)}\text{,} \end{array}$$

(11) $$\begin{array}{c} F^{\mu }=\overset{p}{\underset{i=1}{\sum }}\overset{C}{\underset{c=1}{\sum }}\overset{n_{\mu }}{\underset{p=1}{\sum }}m_{ic}^{\beta }d^{2}\left(x_{ip}^{\mu },v_{cp}^{\mu }\right)\text{,} \end{array}$$

(12) $$\begin{array}{c} m_{ic}=\frac{{\left[\overset{N}{\underset{\mu =1}{\sum }}w_{\mu }\overset{n_{\mu }}{\underset{p=1}{\sum }}d^{2}\left(x_{ip}^{\mu },v_{cp}^{\mu }\right)\right]}^{-1/\beta -1}}{\overset{C}{\underset{c=1}{\sum }}{\left(\overset{N}{\underset{\mu =1}{\sum }}w_{\mu }\overset{n_{\mu }}{\underset{p=1}{\sum }}d^{2}\left(x_{ip}^{\mu },v_{cp}^{\mu }\right)\right)}^{-1/\beta -1}}\text{.} \end{array}$$

### 3.3. View-Level Weighted Fusion Strategy Based on the Belief Function Theory

In the multiview clustering process, the weights of various views are different, which indicates that the reliability of the evidence from various sources is different. Therefore, the membership values of the pattern belonging to different clustering centers are not equally weighted in different views. We use discounting techniques and DS fusion theory to integrate different membership values of the pattern and named it the view-level weighted fusion strategy based on belief function theory. In this method, a classic discounted rule proposed by Shafer in [43] is applied here; the membership values based on the multiple views can be regarded as the evidence that the pattern belongs to all possible classes in the discernment framework. First, we multiply the membership values by the view weights representing reliability. Then, the discounted membership values in different views are fused by a belief function theory. Finally, the clustering results can be obtained. In this section, the membership values are treated as mass values; the view weights are regarded as the discounted factors; and the discounted masses are obtained by equation (13). The discounted masses are regarded as the probability that the pattern belongs to different categories in multiple views. *m*′(Ω) represents the imprecision of the clustering method due to incomplete patterns. In the BTC-MV method, the discounted masses from multiple views are fused by the DS theory, as shown in equation (2). Finally, the clustering results are determined by the maximum belief masses.

(13) $$\begin{array}{c} \left\{\begin{array}{c} m^{\prime }\left(c\right)=w_{\mu }∙m^{\mu }\left(c\right),c\neq \Omega ,\\ m^{\prime }\left(\Omega \right)=1-\sum m^{\prime }\left(c\right). \end{array}\right. \end{array}$$

## 4. Experiment Application

In this section, in order to test the performance of the BTC-MV method, we conduct massive experiments on six data sets with different dimensions from the UCI repository [2]. We divided the attributes of each data set into different groups to satisfy the scenarios of multiple views. Some attributes of this data set are randomly missing to meet the assumption of an incomplete data set. The important information of these well-known data sets, including the number of attributes (*N*_*a*_), classes (*N*_*c*_), instances (*N*_*i*_), and views (*N*_*v*_), is shown in Table 1. These six data sets, where the attributes range from 4 to 16, views range from 2 to 4, classes range from 2 to 7, and the instances range from 150 to 13611, are representative and generic results can be obtained.

In order to justify the performance of the BTC-MV method, the classic imputation methods and clustering methods are combined and compared with the proposed BTC-MV method. The typical methods of estimating missing data include MI [34] and k-nearest neighbors (KNN) [9]. The classic clustering methods used in the comparison experiments include K-means[51], and FCM [52]. According to the number of views, it can be divided into single-view clustering and multiview clustering. Therefore, there are four comparison methods, such as MI-K-means based on single-view clustering (MI-KM), MI-K-means based on multiview clustering (MI-KMVC), KNNI-FCM based on single-view clustering (KNNI-FCM), and KNNI-FCM based on multiview clustering (KNNI-MFCM).

The error rate marked as *R*_*e*_ is used to evaluate the performance of the BTC-MV method. The formula for calculating error rate is *R*_*e*_=*N*_*e*_/*N*, where *N*_*e*_ is the number of the patterns with error clustering results and *N* is the total number of the patterns used to conduct experiments. The experiments are conducted with MATLAB software.

### 4.1. Experiment 1

**Figure 2:**
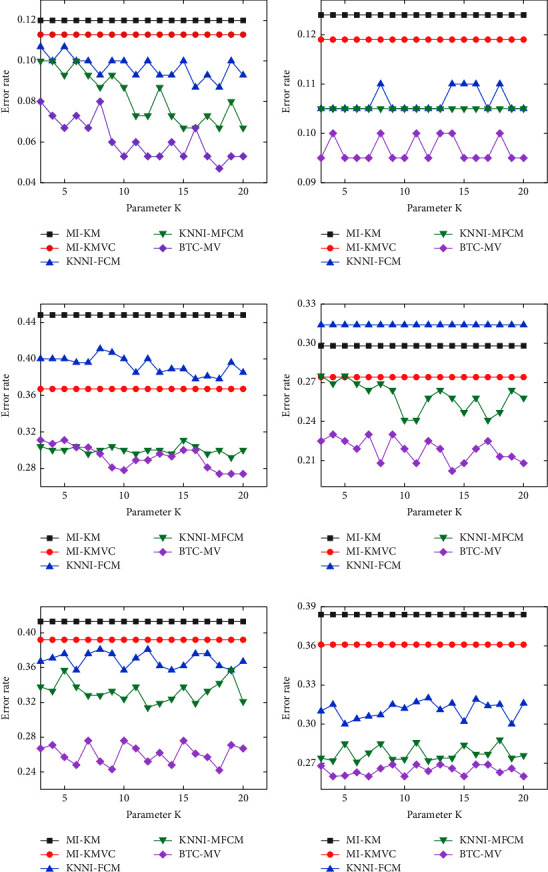
Clustering results of different methods with various parameters *K*. (a) Iris data set. (b) Seeds data set. (c) Heart data set. (d) Wine data set. (e) IS data set. (f) DB data set.

In the methods of MI-KM, MI-KMVC, KNNI-FCM, KNNI-MFCM, and BTC-MV, parameter *K* represents the number of the patterns used to estimate the missing data, and it is one of the main parameters in BTC-MV. In the BTC-MV method, *K* patterns closest to the incomplete data are searched from multiple views with the known attributes. It is worth noting that the parameter *K* influences the precision of the estimations and the performance of the clustering methods. In order to verify the influence of parameter *K* on the clustering methods, numerous experiments are carried out under different *K* values and the comparison results are shown in Figure 2. The error rate of the BTC-MV method varies with the parameter *K*. However, when *K* takes a value from 3 to 20, the error rate of the BTC-MV method fluctuates in an acceptable extent. This result indicates that the BTC-MV method has strong robustness for parameter *K*, which is an advantage of the BTC-MV method in practical classification applications.

### 4.2. Experiment 2

In this experiment, we set each data set to have 10%, 30%, and 50% incomplete patterns, respectively. Moreover, for each incomplete pattern, there are 50% unknown attributes. We compare the performance of the BTC-MV with other clustering methods on six incomplete data sets, which are shown in Tables 2–4. The error rate of the BTC-MV method on different data sets is lower than that of other methods. It may be because the performance of the attribute-level weighted imputation strategy in the BTC-MV method is superior. This imputation method can accurately estimate the missing values because it makes the patterns with high attribute correlation closer to the missing data. So, we can obtain complete data sets with precision estimations and reduce the error rate of the clustering method. It is noteworthy that as the number of missing data increases, the error rate of these methods also increases. This phenomenon indicates that the missing data make the information ambiguous, leading to a degradation in the performance of the clustering methods.

### 4.3. Experiment 3

In this section, we test the influence of the number of unknown attributes in the incomplete patterns. We set each data set to have 30% missing data and each incomplete pattern to have 30%, 50%, and 70% unknown attributes, respectively. We compare the performance of the BTC-MV with other clustering methods on six incomplete data sets, which are shown in Tables 5–7. The results of these experiments indicate that the increase of unknown attributes generally leads to a decrease in clustering performance, as missing data introduce uncertain information. However, compared with other methods, the method of the BTC-MV has superior performance. This experiment further validates the effectiveness and robustness of the BTC-MV method.

## 5. Conclusions

In this paper, the new BTC-MV method is proposed to meet the challenges of incomplete data clustering. The BTC-MV method estimates the unknown attributes by the weighted KNN strategy based on multiple views; the weights are represented by the variance of each attribute, which reflects the importance of the attribute. The attribute-level weighted imputation strategy improves the precision of the estimations. Then, the clustering method based on multiple views is proposed in BTC-MV, and the view weight expresses the reliability of the evidence from different spaces. Therefore, the membership values of the pattern belonging to various categories in multiple views cannot be equally weighted. Finally, in the BTC-MV method, a view-level weighted fusion strategy based on belief function theory is proposed to integrate the evidence from different source spaces. We conducted experiments on six UCI data sets to compare the performance of the BTC-MV method with that of other state-of-the-art methods. The experiment results show that the effectiveness of the BTC-MV method in clustering incomplete patterns.

In the BTC-MV method, the attribute-level weighted imputation strategy makes an important contribution in improving the accuracy of clustering incomplete patterns. However, it is costly to introduce large computations because the distances need to be calculated in the KNN strategy. We will consider using other methods to reduce the computational complexity in future work. In addition, we will also research other methods to optimize the data set in order to obtain superior clustering performance.

## Figures and Tables

**Algorithm 1:**
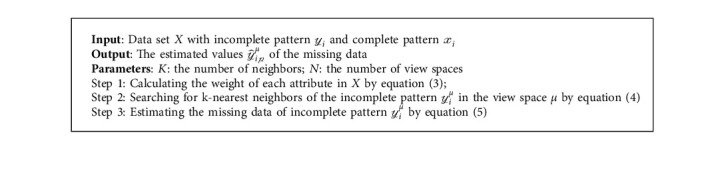
Attribute-level weighted imputation strategy.

**Algorithm 2:**
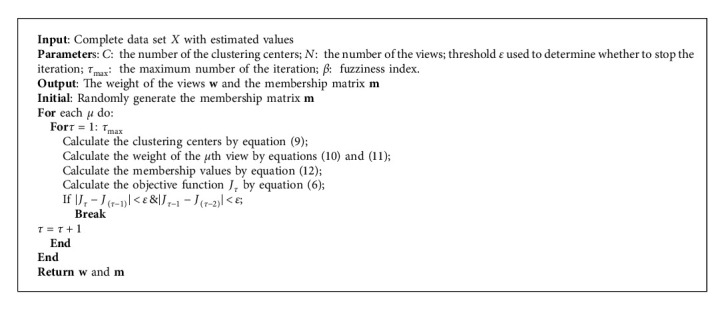
The clustering method based on multiple views.

**Table 1:** Six real data sets from UCI repository.

Data sets	*N *_*a*_	*N*_*v*_	*N *_*c*_	*N *_*i*_
Iris	4	2	3	150
Seeds	7	2	3	210
Wine	13	4	3	178
Heart	13	3	2	270
IS	19	2	7	2310
DB	16	2	7	13611

**Table 2:** Experiments on six data sets in the UCI repository with 10% missing data.

	Iris	Seeds	Heart	Wine	IS	DB
MI-KM	0.12	0.12	0.43	0.33	0.41	0.38
MI-KMVC	0.11	0.12	0.36	0.27	0.38	0.36
KNNI-FCM	0.10	0.10	0.36	0.31	0.34	0.32
KNNI-MFCM	0.09	0.10	0.30	0.25	0.30	0.28
BTC-MV	**0.07**	**0.09**	**0.27**	**0.21**	**0.24**	**0.26**

In each data set, the bold value is the lowest error rate.

**Table 3:** Experiments on six data sets in the UCI repository with 30% missing data.

	Iris	Seeds	Heart	Wine	IS	DB
MI-KM	0.13	0.13	0.43	0.37	0.43	0.41
MI-KMVC	0.11	0.12	0.37	0.30	0.39	0.38
KNNI-FCM	0.11	0.11	0.37	0.31	0.36	0.33
KNNI-MFCM	0.10	0.10	0.31	0.26	0.31	0.30
BTC-MV	**0.07**	**0.10**	**0.27**	**0.22**	**0.26**	**0.28**

In each data set, the bold value is the lowest error rate.

**Table 4:** Experiments on six data sets in the UCI repository with 50% missing data.

	Iris	Seeds	Heart	Wine	IS	DB
MI-KM	0.13	0.14	0.45	0.38	0.42	0.42
MI-KMVC	0.11	0.12	0.40	0.33	0.45	0.40
KNNI-FCM	0.11	0.11	0.39	0.31	0.39	0.34
KNNI-MFCM	0.11	**0.10**	0.31	0.28	0.33	0.31
BTC-MV	**0.08**	**0.10**	**0.29**	**0.24**	**0.27**	**0.30**

In each data set, the bold value is the lowest error rate.

**Table 5:** Experiments on six data sets with 30% unobservable attributes.

	Iris	Seeds	Heart	Wine	IS	DB
MI-KM	0.11	0.13	0.42	0.33	0.42	0.43
MI-KMVC	0.09	0.11	0.38	0.27	0.38	0.39
KNNI-FCM	0.10	0.11	0.40	0.30	0.324	0.32
KNNI-MFCM	0.09	0.10	0.30	0.25	0.28	0.28
BTC-MV	**0.06**	**0.10**	**0.26**	**0.22**	**0.26**	**0.27**

In each data set, the bold value is the lowest error rate.

**Table 6:** Experiments on six data sets with 50% unobservable attributes.

	Iris	Seeds	Heart	Wine	IS	DB
MI-KM	0.13	0.13	0.43	0.37	0.43	0.41
MI-KMVC	0.11	0.12	0.37	0.30	0.39	0.38
KNNI-FCM	0.11	0.11	0.37	0.31	0.36	0.33
KNNI-MFCM	0.10	0.10	0.31	0.26	0.31	0.30
BTC-MV	**0.07**	**0.10**	**0.27**	**0.22**	**0.26**	**0.28**

In each data set, the bold value is the lowest error rate.

**Table 7:** Experiments on six data sets with 70% unobservable attributes.

	Iris	Seeds	Heart	Wine	IS	DB
MI-KM	0.12	0.13	0.42	0.34	0.44	0.42
MI-KMVC	0.10	0.12	0.39	0.31	0.37	0.40
KNNI-FCM	0.10	0.12	0.41	0.32	0.31	0.33
KNNI-MFCM	0.10	0.12	0.31	0.26	0.28	0.31
BTC-MV	**0.06**	**0.10**	**0.28**	**0.24**	**0.27**	**0.29**

In each data set, the bold value is the lowest error rate.

## Data Availability

The data sets used in this proposal are extracted from the University of California Irvine machine learning repository.
